# Lipid from electronic cigarette-aerosol both with and without nicotine induced pro-inflammatory macrophage polarization and disrupted phagocytosis

**DOI:** 10.1186/s12950-023-00367-6

**Published:** 2023-11-17

**Authors:** Mizanur Rahman, Shanzina Iasmin Sompa, Micol Introna, Swapna Upadhyay, Koustav Ganguly, Lena Palmberg

**Affiliations:** Unit of Integrative Toxicology, Institute of Environmental Medicine, Karolinska Institutet, 171 77 Stockholm, Sweden

**Keywords:** Electronic cigarette, Macrophage, M1, M2, Lipid, Malondialdehyde, Phagocytosis, *E. coli*

## Abstract

Clinical cases and experimental evidence revealed that electronic cigarettes (ECIG) induce serious adverse health effects, but underlying mechanisms remain to be fully uncovered. Based on recent exploratory evidence, investigating the effects of ECIG on macrophages can broadly define potential mechanisms by focusing on the effect of ECIG exposure with or without nicotine. Here we investigated the effect of ECIG-aerosol exposure on macrophages (MQ) phenotype, inflammatory response, and function of macrophages.

MQ were cultured at air liquid interface and exposed to ECIG-aerosol. Oxidative stress was determined by reactive oxygen species (ROS), heat shock protein 60 (HSP60), glutathione peroxidase (GPx) and heme oxygenase1 (HMOX1). Lipid accumulation and lipid peroxidation were defined by lipid staining and level of malondialdehyde (MDA) respectively. MQ polarization was identified by surface expression markers CD86, CD11C and CD206 as well as pro-inflammatory and anti-inflammatory cytokines in gene and protein level. Phagocytosis of *E. coli* by MQ was investigated by fluorescence-based phagocytosis assay.

ECIG-aerosol exposure in presence or absence of nicotine induced oxidative stress evidenced by ROS, HSP60, GPx, GPx4 and HMOX1 upregulation in MQ. ECIG-aerosol exposure induced accumulation of lipids and the lipid peroxidation product MDA in MQ. Pro-inflammatory MQ (M1) markers CD86 and CD11C but not anti-inflammatory MQ (M2) marker CD206 were upregulated in response to ECIG-aerosol exposure. In addition, ECIG induced pro-inflammatory cytokines IL-1beta and IL-8 in gene level and IL-6, IL-8, and IL-1beta in protein level whereas ECIG exposure downregulated anti-inflammatory cytokine IL-10 in protein level. Phagocytosis activity of MQ was downregulated by ECIG exposure. shRNA mediated lipid scavenger receptor ‘CD36’ silencing inhibited ECIG-aerosol-induced pro-inflammatory MQ polarization and recovered phagocytic activity of MQ.

ECIG exposure alters lung lipid homeostasis and thus induced inflammation by inducing M1 type MQ and impair phagocytic function, which could be a potential cause of ECIG-induced lung inflammation in healthy and inflammatory exacerbation in disease condition.

**Supplementary Information:**

The online version contains supplementary material available at 10.1186/s12950-023-00367-6.

## Introduction

The trends of using electronic cigarettes (ECIG) are increasing. First generation of ECIG was introduced in 2003 and became available commercially in 2007 in the US and spread through the countries [1]. The use of ECIG is dramatically increasing and it is predicted that it will supersede traditional smoking in next ten years [2]. Although ECIG was expected as a safer alternative than the traditional smoking, in the US by the mid of February 2020, a total 2807 ECIG users got severe lung injury, hospitalized and 68 deaths were confirmed [3, 4]. The emergence of a case report from 98 patients related to lung illness reported various clinical symptoms and toxicity [4]. The patients reported respiratory symptoms, cough, chest pain, gastrointestinal symptoms, and constitutional symptoms. Clinically, 100% of patients with vaping associated lung injury experience constitutional symptoms such as fever, chills, and weight loss, whereas respiratory symptoms were present in 97% of patients [4]. In addition, bilateral infiltrates on chest X-ray were reported. Mild and non-specific inflammation as well as foamy or lipid laden macrophages (MQ) were identified [4–7]. Moreover, mice studies revealed that chronic ECIG-aerosol exposure downregulates MQ-mediated innate immunity [7]. Most importantly, the study showed lipid dysregulation in response to ECIG-aerosol exposure. Disrupted lipid homeostasis in lungs may contribute to the pathogenesis of COPD [8]. In some studies ECIG-induced toxicity and inflammatory responses were determined as an nicotine independent phenomena [7, 9] whereas nicotine dependent TLR2 reduction was also reported [10]. Chronic exposure to nicotine containing ECIG was reported to be associated with development of COPD in mice [11]. Use of ECIG might cause an unknown vaping related lung disease as well as potential cause of cardiovascular diseases after chronic exposure [12, 13]. First comprehensive study in human volunteers that examined acute vascular impact against ECIG has been reported [13]. The study identified that ECIG induced arterial stiffness, which is an early indicator of cardiovascular disease [13]. According to accumulated data from experimental studies, ECIG induce inflammatory responses, oxidative stress, cell death and DNA damage as well as reduced phagocytosis of bacteria by macrophages [9, 14]. In the proximal airways and alveoli, MQ are the specialized resident immune cells that encounter and clean up inhaled particles like pollutants or antigens and thus regulate cellular homeostasis [15, 16]. Alteration in function of MQ in airways can lead to the pathogenesis of lung disease such as COPD and increase susceptibility to infection such as pneumonia [15, 16].

In ECIG-aerosols, around 7,000 different chemicals were identified [9]. We found in an in vitro study that ECIG with or without nicotine induced pro-inflammatory effects and DNA methylation [17]. In addition, in a cross-sectional study, we recently found significantly higher prevalence of cough and mucus production among ECIG users in comparison to non-ECIG users [18].

Although the recommendations of the European Respiratory Society task force on ECIG research included the need for identification of molecular patterns as well as studies characterizing the health effects and toxicology of ECIG flavorings [5], the underlying mechanism of ECIG-induced adverse health effects are not fully uncovered. Despite the commonality of lipid accumulation in lung of ECIG users, the physiological importance of accumulation of lipid and whether such phenomenon can be recapitulated in experimental systems remain to be elucidated. More specifically, information on the effect of ECIG on the immune cell (MQ) function is still incomplete. Very limited data is available comparing the potential effects of ECIG exposure on alveolar MQ in presence and absence of nicotine with the same flavor. In the current study, we investigated the effect of ECIG in presence or absence of nicotine on MQ polarization, inflammatory and oxidative stress response as well as phagocytosis activities of MQ in connection with lipid homeostasis.

## Materials and methods

### Cell culture 

Buffy coat was collected from Karolinska University hospital, Sweden, and monocytes were isolated from the buffy coats by a negative selection kit (Stem cell technologies, UK). Isolated monocytes were stimulated with 50 ng/ml of GM-CSF (Immunotools, Germany) for 5 days to differentiate into macrophages. Macrophage differentiation was confirmed by light microscopic visual observation and CD11B staining. The differentiated macrophages were trypsinized and seeded in 12 wells transwell inserts with 0.4 µm pore size (Corning, Sigma Aldrich, Sweden) following earlier protocol for THP1 derived macrophages [19]. After overnight incubation, cell culture media was removed from the apical side to start culturing at air–liquid interface (ALI) and the basal side was filled with the RPMI complete media with 10% FBS and 1% penicillin /Streptomycin.

### ECIG exposure

MQ were exposed to ECIG-aerosol according to the earlier established protocol [17, 20]. According to the earlier study, we selected the ECIG flavor 2 (ripe strawberry, sweet apples and kiwi) which was more toxic than the flavor 1 [17]. For this study, the difference between ECIG with nicotine (3 mg/ml) and without nicotine was just nicotine. The detail, characterization of these ECIG liquid was published in our earlier study [17].

Shortly, MQ in 12 well plates were placed in a glass jar with 3L desiccator volume, maintained at 37 °C and humidity above 70% and allowed to equilibrate for 10–15 min. An air-tight pre-heated glass syringe was used to repeatedly collect 40 ml (representing one puff) of ECIG-aerosol and injected it into the desiccator. Ten puffs were injected to mimic one vaping session. The inlet tube contained multiple sidewise apertures for an even spread of the ECIG-aerosol within the desiccator. The macrophages cultured at ALI were exposed to ECIG-aerosol or filtered air for 15 min, where after they were transferred to a cell incubator (37 °C, 60% humidity and 5% CO_2_) for 1 h (h) until the next exposure session. MQ were exposed to ECIG-aerosol total 3 times and 10 puffs in each exposure with 1 h between each exposure. Following completion of 3 exposures, the MQ were incubated for various time points depending on the readout indicated below.

### Cell viability and apoptosis assay

To evaluate whether ECIG-aerosol exposure induce apoptosis or cell death, LDH assay (Thermofisher, Sweden) and annexin V assay (BD Bioscience, USA) were performed following manufacturer protocol. Shortly, MQ were exposed to ECIG-aerosol with or without nicotine and incubated for 18 h. After the incubation, cell culture supernatant was used for LDH assay and cells were stained with annexin V for apoptosis measurement by flow cytometry.

### ROS measurement

Environmental insults including cigarette smoke induce oxidative stress and generate reactive oxygen species (ROS). Therefore, we evaluated the level of ROS in response to ECIG in the presence or absence of nicotine. After the 3^rd^ exposure to ECIG-aerosol, MQ were incubated for 2 h. After the incubation, basal media was removed, and both apical and basal side of the insert were washed three times with PBS. Five µM of Cell ROX reagent (Thermofisher, Sweden) in RPMI media was added at both basal (500 µl) and apical side (500 µl) of the insert. After 30 min of incubation, cells were washed 3 times with PBS, trypsinized, resuspended in PBS and collected into flow cytometry tubes. In the presence of reactive oxygen species (ROS), CellROX reagents generate fluorescence which is proportional to ROS level. The total ROS level was determined by flow cytometry, and median fluorescence intensity (MFI) was presented as the level of ROS generation.

### Phospholipid measurement

According to the manufacturer protocol (Abcam, UK), levels of phospholipids in ECIG liquid and ECIG vapor condensate were measured by phospholipid assay kit.

### Flow cytometry

MQ were incubated for 18 h after the 3^rd^ exposure to ECIG-aerosol. MQ were trypsinized and incubated with Fc blocker for 10 min on ice. After the incubation with Fc blocker, the MQ were incubated for 30 min on ice with the antibodies, specific to M1 MQ markers (PerCp5.5-labeled CD86 and PE-labeled CD11C), M2 MQ marker (FITC-labeled CD206), TLR2 (BV-421-labeled), TLR4 BV711-labeled) and lipid scavenger receptor ‘CD36’ (PE labeled-CD36) (BD bioscience, US). Cells were washed 3 times with PBS, resuspended in fresh PBS and the expression of these cell surface markers was investigated by flow cytometry using single or multi color fluorochrome. In the muti color assay, compensation was performed by compensation beads (BD bioscience, US) to avoid spectral overlap. The raw data from flow cytometry was analyzed by Flow Jo software and the expression level was presented as median fluorescence intensity.

### Malondialdehyde measurement

According to the manufacturer instruction, Lipid peroxidation product malondialdehyde (MDA) was measured from the cell lysate using MDA assay kit (Sigma Aldrich, Sweden). In order to detect MDA-modified protein, MQ was incubated as above with FITC-labeled MDA antibodies (antibodies against MDA-modified protein, Abcam UK) and as above, the level of MDA-modified proteins was investigated by flow cytometry.

### RT-qPCR

After the 3^rd^ exposure to ECIG-aerosol, MQ were incubated for 6 h, and mRNA was extracted from the MQ by RNA extraction mini kit (Qiagen, Germany). cDNA was synthesized from 300 ng of RNA by cDNA synthesis kit (Applied biosystem, Germany) and 100 ng of cDNA was used in each reaction of RT-qPCR. PCR amplification primers were selected according to the earlier study [17]. Housekeeping gene glyceraldehyde 3-phosphate dehydrogenase (GAPDH) was used as an internal control. The expression levels of oxidative stress related genes glutathione peroxidase (*GPx),* glutathione peroxidase *(GPx4)* and *HOMOX1* as well as inflammatory cytokines genes *IL-6, TNF-α, IL-12, IL-1β* and *IL-10* were calculated by delta-delta CT methods.

### ELISA

After the 3^rd^ exposure, cell culture media was collected after 18 h of incubation. Secreted cytokines including IL-6, TNF-α, IL1-β, IL-8, IL-12A and IL-10 levels were measured from the culture supernatant by ELISA duoset (Biotechne, UK). In addition, cell lysates were prepared after 6 h of 3^rd^ exposures and level of HSP60 was measured from the cell lysates using ELISA duoset (Biotechne, UK).

### Lipid accumulation assay

Lipid accumulation was measured by flow cytometry and microscopy. After ECIG exposure, according to the manufacturer's protocol, cells were stained with lipid staining reagent BODIPY (Sigma Aldrich, Sweden) and lipid accumulation was investigated by flow cytometry and confocal microscopy. Median fluorescence intensity was presented as the level of expression from Flow cytometry measurement and microscopic image was presented after developed by image J software.

### CD36 silencing

According to the manufacturer protocol (Santa Cruz, Germany), CD36 was silenced with shRNA. In brief, differentiated MQ were cultured with serum free medium in 6 wells plate and transfected with CD36 shRNA or with a control shRNA. After 6 h of transfection, 10% FBS was added to the culture medium. After 72 h, more than 70% reduced expression of CD36 was confirmed at gene and protein level by qRT-PCR and flow cytometry, respectively.

### Phagocytosis

According to the manufacturer's protocol (Vybrant™ Phagocytosis Assay Kit, Thermo Fisher, Sweden), phagocytosis of *E. coli* particles (FITC labeled) by MQ was investigated. In short, MQ-cultured at ALI were exposed to ECIG-aerosol as described above. After overnight incubation, apical side of the insert was washed with RPMI media and *E. coli* particles in 100µl of media was added on the top of the MQ. After 3 h of incubation, MQ were trypsinized, transferred into 96 wells plates and the plates were read at 485 excitation wavelengths by microplate reader (Bioteknik, US). Percentage of phagocytosis was calculated and compared between control and exposed condition. As described above, MQ were exposed to ECIG and incubated for 18 h after the 3^rd^ exposure and stained with CD35 (complement receptor1) and CD64 (Fc receptor) antibodies (BD bioscience, US) to detect surface expression. The expression of these cell surface markers was determined by flow cytometry, and the expression level was presented as median fluorescence intensity (MFI).

### Statistical analysis

Each experiment was performed with MQ from 3 donors (*N* = 3), and technical replicates *n* = 2–3 from each donor. The results were expressed as median and interquartile ranges (25th–75th percentiles) followed by non-parametric statistical analysis. Within each group, the comparisons between control and ECIG exposure with or without nicotine were performed by the Friedman test and followed by Wilcoxon signed-rank test. In all statistical tests, the difference with a *P* value ≤ 0.05 was considered statistically significant. Statistical significance in comparison to clean filtered air (sham) to all treatment condition expressed with * and between exposure with or without nicotine was expressed with #. Pearson two tailed analysis was performed to determine correlation between lipid accumulation and dependent variables and correlation matrix to evaluate the correlation between each variable. All the data were analyzed using the GraphPad Prism 8.30 software.

## Results

### Cell viability and apoptosis

ECIG-aerosol exposure with or without nicotine did not affect viability of the MQ (data not shown). In the presence or absence of nicotine, ECIG did not induce apoptosis. Although, there was a trend of minimal induction of apoptosis in response to ECIG with nicotine, but the effect was not significant statistically (Supp. Fig. 1).

### ECIG induced oxidative stress

**Fig. 1:**
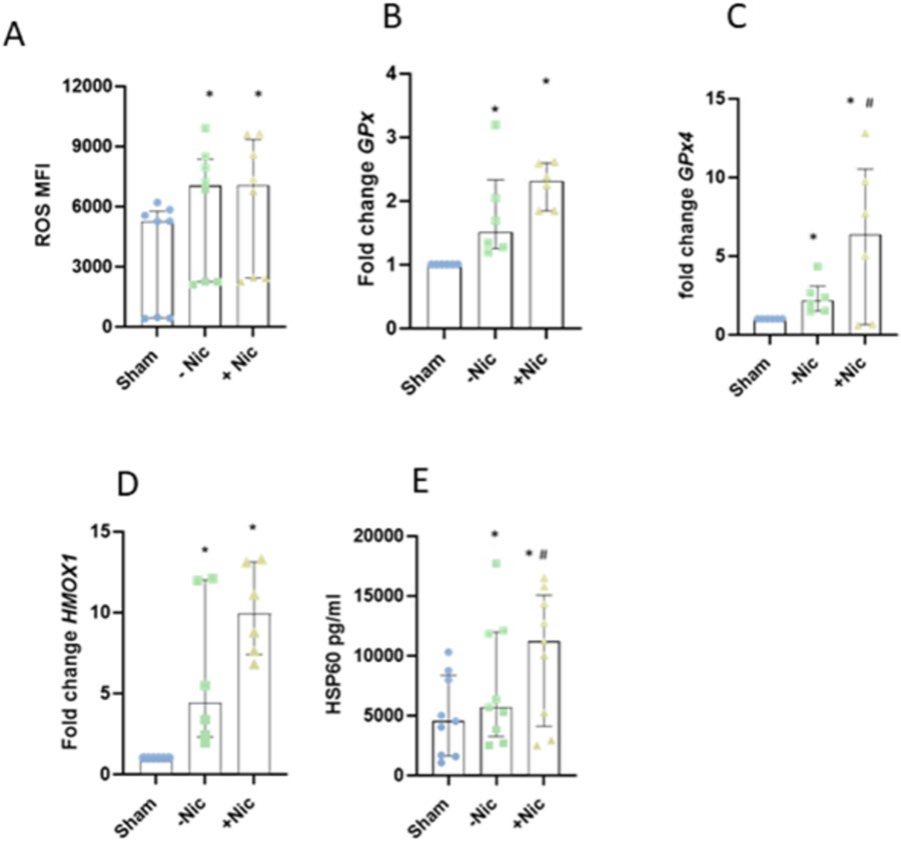
ECIG-aerosol expposure ndued oxidative stress in presence or absence of nicotine. Reactive oxygen species prodution was induced in response to ECIG exposure (**A**). Anti-oxidant (GPx) and lipid peroxidanation specific antioxidant (*GPx4)* was upregulated by ECIG with or without nicotine but GPx was increased promiarily in prsesence of nictoine (**B**) and (**C**). *HMOX1* in gene level was increaed in response ECIG exposure (**D**). HSP60 expression was upregulated in response to ECIG expopsure both with nicotine and without nicotine but primarily with nicotine (**E**). Statistical significance (*p* ≤ 0.05) between Sham and exposure (nicotine or without nicotine) condition was indicated as *. Statistical significance between nicotine and without nicotine was indicated as #

Oxidative stress was assessed by measuring the level of ROS production. The level of ROS generation was increased significantly in response to ECIG-aerosol exposure both with and without nicotine (Fig. 1A). The levels of oxidative stress related genes *GPx, GPx4* and *HMOX1* (Fig. 1B-D) were increased significantly in response to ECIG vapor both with and without nicotine. *GPx4* increased primarily in the presence of nicotine (Fig. 1C). In addition, stress related protein HSP60 was increased significantly in response to ECIG-aerosol exposure both with and without nicotine but primarily in presence of nicotine (Fig. 1E).

### ECIG-aerosol induced lipid accumulation and lipid peroxidation

**Fig. 2:**
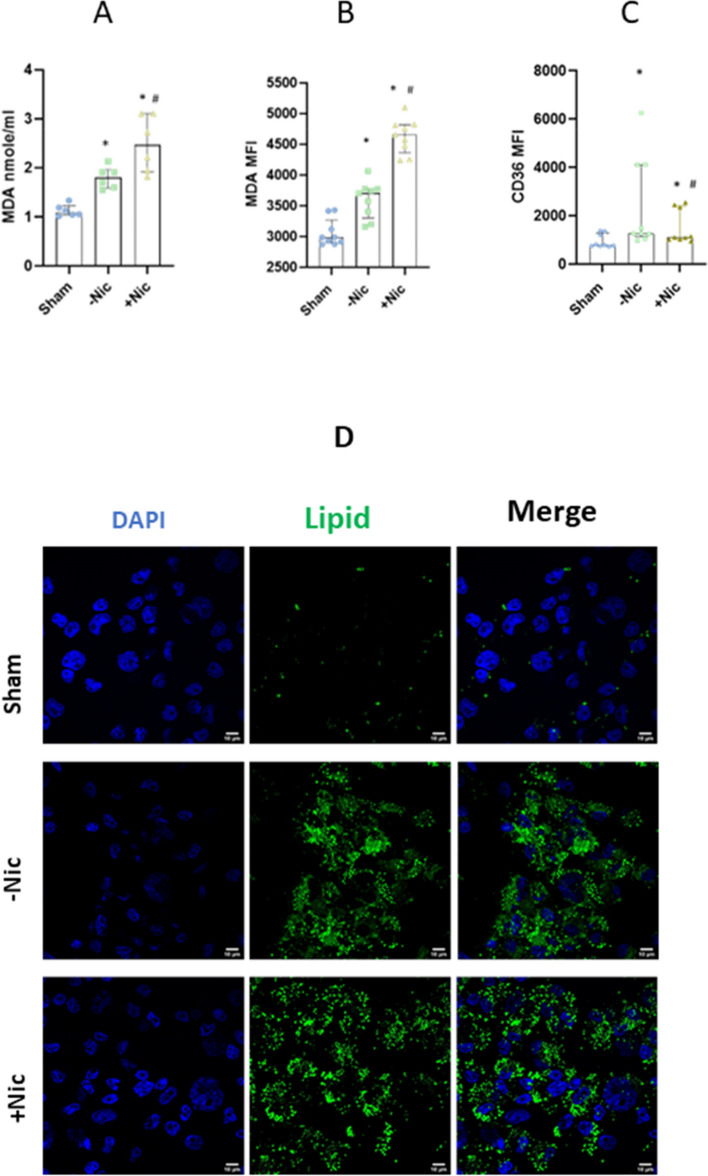
ECIG-aerosol exposure induced lipid peroxidation and lipid accumulation. ECIG induced lipid peroxidation product malondialdehyde (MDA) (**A**) and MDA-modified proteins in MQ (**B**). Lipid scavenger receptors CD36 (**C**) and lipid accumulation were increased in MQ (**D**). Statistical significance (*p* ≤ 0.05) between Sham and exposure (nicotine or without nicotine) condition was indicated as *. Statistical significance between nicotine and without nicotine was indicated as #

Phospholipids were detected in both ECIG liquid and ECIG vapor condensate (Supp. Fig. 2). Next, lipid peroxidation was measured in ECIG-aerosol exposed-MQ, both ECIG-aerosol with and without nicotine, but primarily with nicotine induced lipid peroxidation product MDA (Fig. 2A). Exposure to ECIG-aerosol both with and without nicotine, but significantly more with nicotine induced MDA-modified protein (Fig. 2B). Furthermore, lipid scavenger receptor (CD36) expression was increased in response to ECIG- aerosol both with and without nicotine, but primarily without nicotine (Fig. 2C). In addition to lipid scavenger receptor, lipid accumulation was increased significantly in ECIG-aerosol (both with and without nicotine)-exposed MQ (Fig. 2D).

### ECIG-aerosol induced pro-inflammatory macrophage polarization

**Fig. 3:**
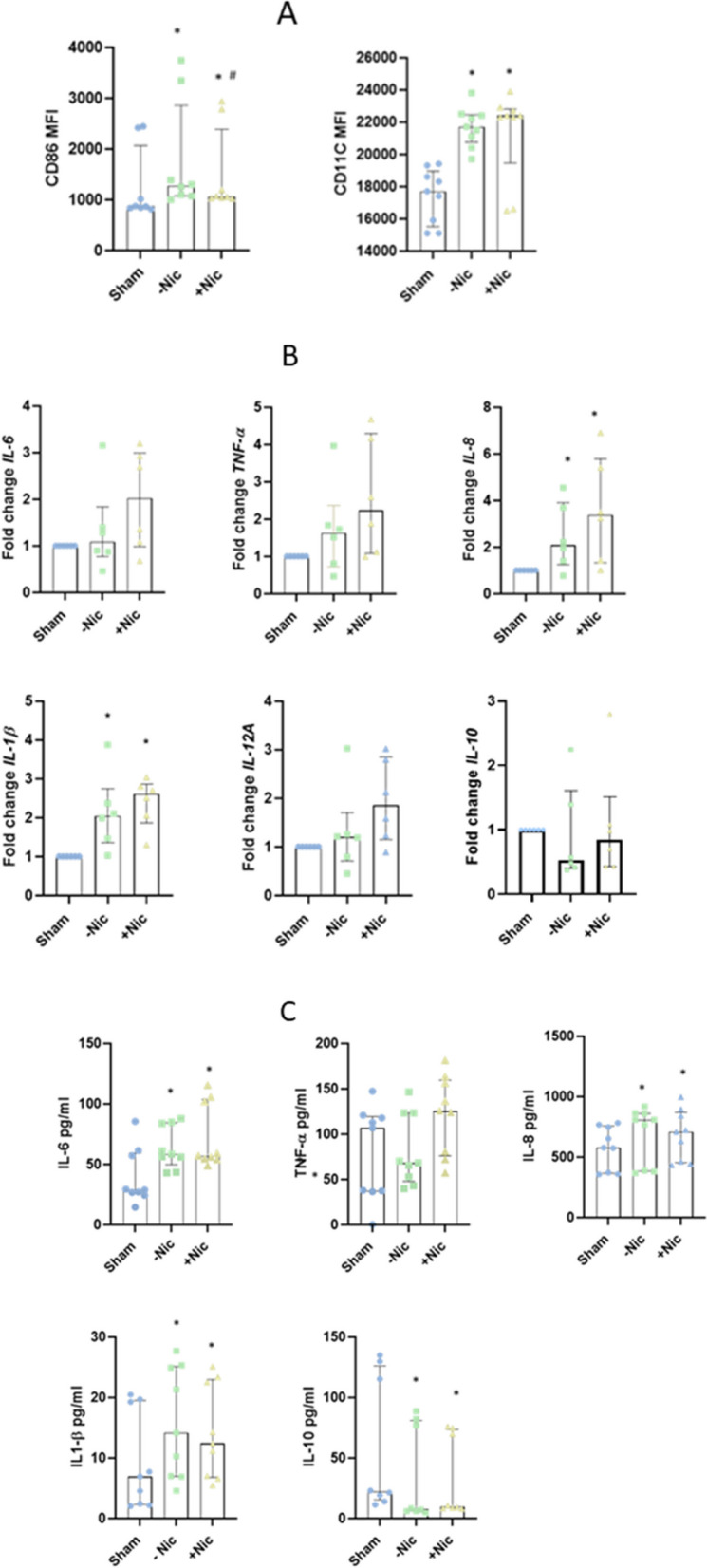
Effects of ECIG-aerosol exposure on MQ polarization and inflammatory response in MQ cultured at air–liquid interface. ECIG-aerosol induced MQ polarization markers CD86 and CD11c (**A**). The ECIG-aerosol exposure induced levels of inflammatory cytokines in gene level (**B**) and in secreted protein level (**C**). Statistical significance (*p* ≤ 0.05) between Sham and exposure (nicotine or without nicotine) condition was indicated as * and between nicotine and without nicotine was indicated as #

ECIG-aerosol exposure resulted in increased expression of pro-inflammatory M1 MQ polarization markers ‘CD86’ and ‘CD11C’. M1 markers were induced significantly both in presence and absence of nicotine compared to sham, whereby CD86 expression was significantly higher in absence of nicotine compared to nicotine containing ECIG vapor (Fig. 3A). However, ECIG-aerosol exposure did not induce M2 macrophage marker ‘CD206’ significantly (Supp. Fig. 3). The expression of pro-inflammatory cytokines *IL-8* and *IL-1 beta* was increased significantly at gene level whereas the levels of *TNF, IL-6, IL-12A* and *IL-10* were not affected at gene levels after exposure to ECIG-aerosol (Fig. 3B). In addition, secretion of inflammatory cytokines IL-6, IL-8 and IL-1 beta was increased significantly against exposure to ECIG-aerosol both in presence and absence of nicotine (Fig. 3C). However, the release of the anti-inflammatory cytokine IL-10 was reduced significantly in presence or absence of nicotine (Fig. 3C).

### CD36 silencing inhibited lipid accumulation and MQ polarization

**Fig. 4:**
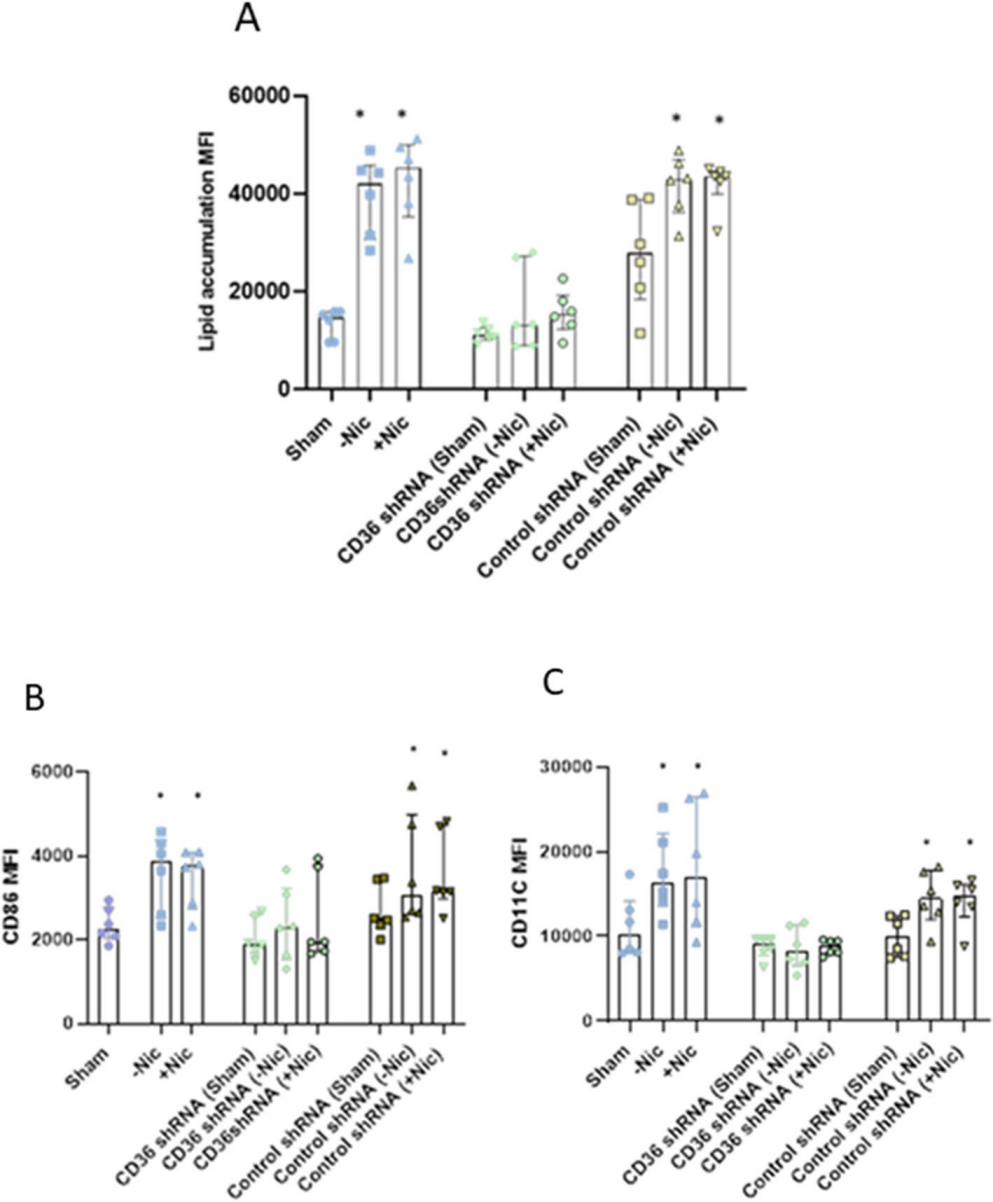
CD36 silencing inhibited lipid accumulation and MQ polarization. CD36 silencing inhibited lipid uptake in MQ (**A**). M1 type MQ polarization markers CD86 (**B**) and CD11C (**C**) expression was reduced in CD36-silenced MQ. Statistical significance (*p* ≤ 0.05) between Sham and exposure (nicotine or without nicotine) condition was indicated as * in each group (Control group, CD36 shRNA group and control shRNA group). CD35 and CD64 expression was downregulated in ECIG-aerosol-exposed MQ

Silencing of CD36 suppressed ECIG-aerosol -induced lipid accumulation in MQ (Fig. 4A). In addition, ECIG-aerosol-induced expressions of MQ polarization markers ‘CD86’ and ‘CD11C’ were inhibited significantly by CD36 silencing (Fig. 4B-C).

### ECIG exposure induced TLR4 but not TLR2 expression

**Fig. 5:**
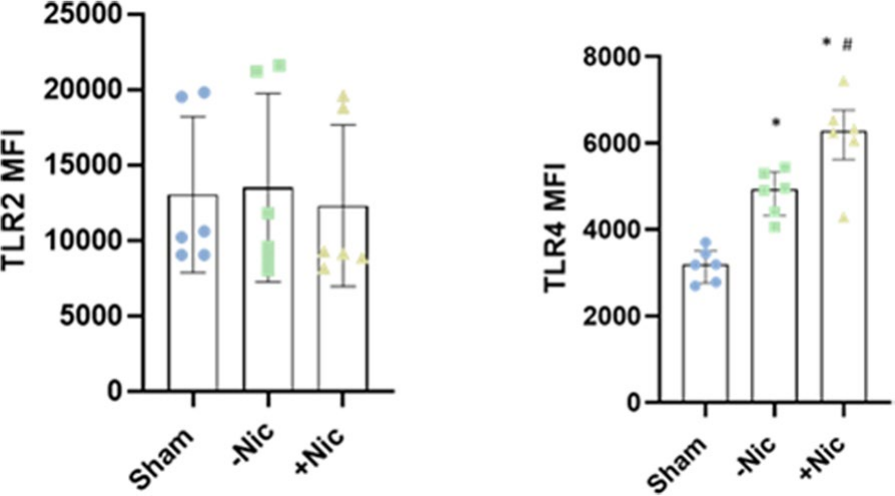
ECIG-aerosol exposure induced TLR4 expression. In presence or absence of nicotine but primarily in presence of nicotine, ECIG-aerosol induced surface expression of TLR4 whereas TLR2 expression was not affected significantly. Statistical significance (*p* ≤ 0.05) between Sham and exposure (nicotine or without nicotine) condition was indicated as *. Statistical significance between nicotine and without nicotine was indicated as #

In comparison to air (sham) exposure, ECIG-aerosol exposure with or without nicotine did not affect the expression of TLR2 but induced the expression of TLR4 on MQ (Fig. 5).

### ECIG-aerosol exposure induced decrease in phagocytosis of *E.coli*

**Fig. 6:**
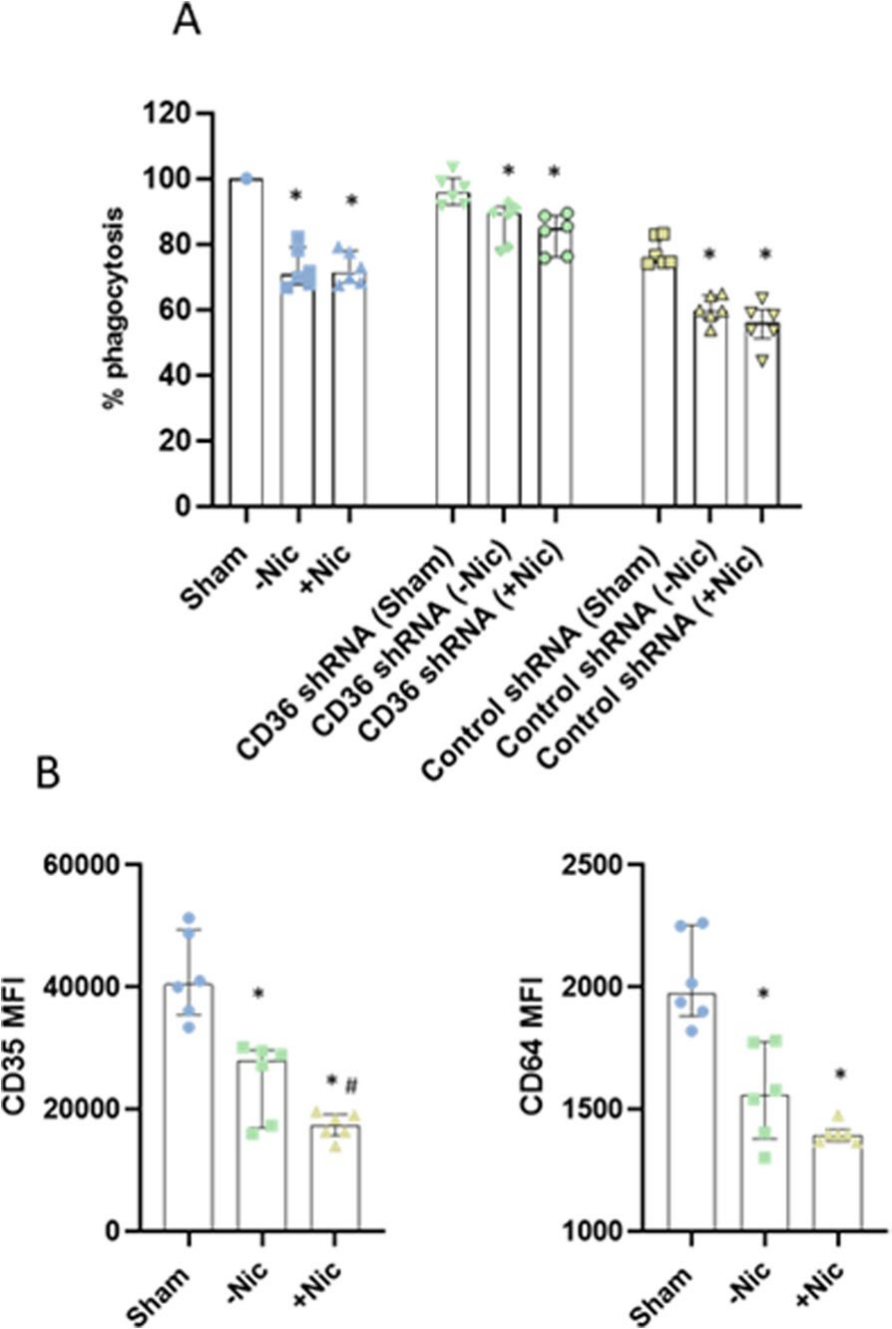
ECIG-aerosol exposure inhibited phagocytosis of *E.coli.* MQ reduced phagocytosis of E. coli. after exposed to ECIG-aerosol (**A**) Statistical significance (*p* ≤ 0.05) between Sham and exposure (nicotine or without nicotine) condition was indicated as * in each group Control group, CD36 shRNA group and control shRNA group). CD35 and CD64 expression was downregulated in ECIG-aerosol-exposed MQ (**B**) and (**C**). Statistical significance (*p* ≤ 0.05) between Sham and exposure (nicotine or without nicotine) condition was indicated as *

ECIG-aerosol exposure both with and without nicotine, affected phagocytosis activity of MQ. In comparison to filtered air (sham), ECIG-aerosol-exposed MQ engulfed *E.coli* particles in reduced levels (Fig. 6A). CD36 silencing recovered phagocytosis activity in ECIG-aerosol exposed MQ (Fig. 6A). Furthermore, after ECIG-aerosol exposure, a reduced expression of phagocytosis receptors CD35 and CD64 was noticed both after exposure to ECIG with or without nicotine, but regarding CD35 expression, primarily with nicotine (Fig. 6B).

### Lipid accumulation is associated with MQ phenotype, phagocytosis, lipid peroxidation and inflammatory markers

**Fig. 7:**
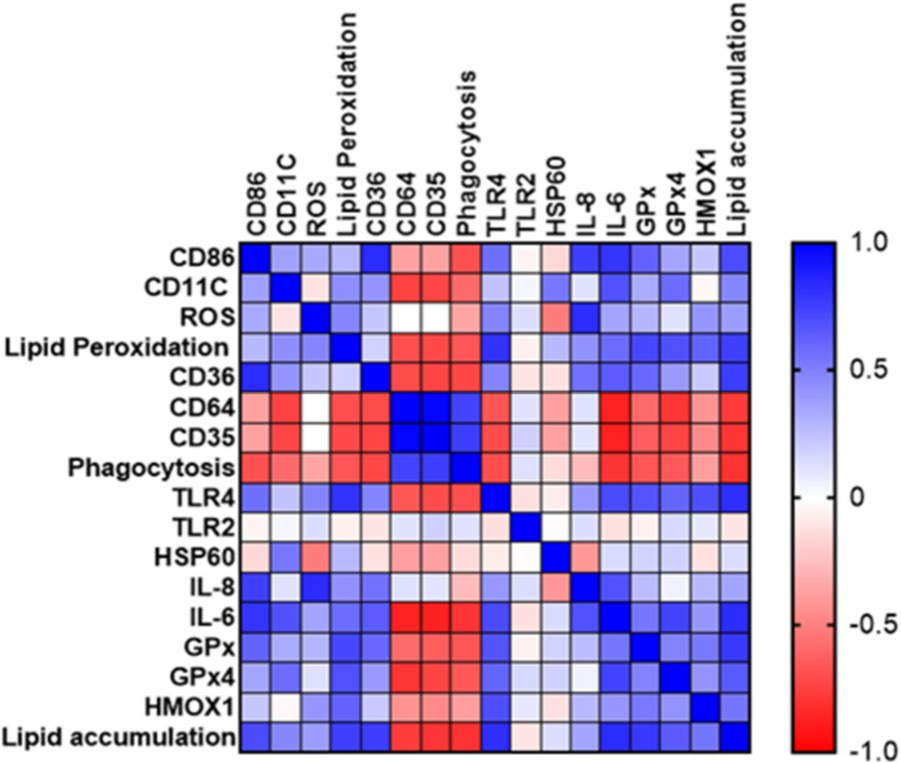
Matric correlaltion. Heatmap shows the positve or negative colleration between each bio-markers

Lipid accumulation was positively correlated with CD86, CD11C, CD36, lipid peroxidation, TLR4, IL-6, *GPx, GPx4, HMOX1* and negatively correlated with CD35, CD64 and phagocytosis (Supp. Fig. 4 and Supp. Table 1). Correlation between lipid accumulation and ROS, HSP60, TLR2 or IL-8 was not statistically significant (Supp. Fig. 4 and Supp Table 1). Correlation between each variable was presented in the heatmap (Fig. 7).

## Discussion

In this study, we investigated the effect of ECIG vapor with or without nicotine on MQ polarization and its function on oxidative stress, inflammation, and phagocytosis in connection with lipid homeostasis in an in-vitro experimental set-up, using peripheral blood monocyte derived MQ cultured at ALI. Our data highlights the importance of MQ in lipid homeostasis and overall immune function of MQ in lungs in response to ECIG exposure. ECIG-induced generation of ROS in airway epithelial cells have been reported in earlier studies [21]. Our data determined that ECIG-induced ROS generation in MQ is nicotine independent. Induction of GPx expression is an important step to reduce oxidative stress to balance redox homeostasis [22]. GPx4 is induced in response to lipid peroxidation. ECIG with or without nicotine, but primarily with nicotine induced *GPx4* at gene level, indicating that ECIG exposure in presence of nicotine increases higher level of cellular lipid peroxidation. Recent investigation in a mice model showed that chronic exposure to ECIG reduce the level of GPx [23], indicating that chronic exposure to ECIG-aerosol potentially imbalance the redox state and induce inflammation, thus potentially play role in pathogenesis of different diseases such as COPD and fibrosis.

Among all HSP, HSP60 is more conserved in prokaryotic and eukaryotic cells and HSP60 is also involved in inflammation. We determined in our earlier study that HSP60 activates immune cells and thus induces inflammation [24]. ECIG-induced HSP60 upregulation potentially facilitates MQ activation and induces inflammation in the airways. Since GPx4 induction indicates lipid peroxidation, therefore, we measured the lipid peroxidation product malondialdehyde (MDA). Our result determined that ECIG exposure with or without nicotine, but primarily with nicotine induces the lipid peroxidation product MDA, which is consistent with the GPx4 induction. Our earlier study showed that MDA-modified human serum albumin induces M1 MQ polarization [25] which indicates that ECIG-induced lipid peroxidation is a potential cause of M1 MQ polarization. Vitamin E acetate (VEA) of ECIG as a potential causative factor for oxidative damage-induced lung injury [26–28]. VEA exposure increased the level of MDA in a murine model [28], which was suggested as a pathway for systemic inflammation in the murine model of EVALI. Since vitamin E is a lipid soluble antioxidant, the study speculated that VEA can be converted to vitamin E by alveolar MQ. Alveolar macrophage forms the first line of defense against pollutants and microorganisms and uses a variety of pattern recognition and scavenger receptors to sense and phagocytose pathogens [29]. MQ are known phagocytic cell type, but dynamic factors in MQ including MQ phenotype, difference in maturation and tissue migration have been identified. Lipid regulation has a potential effect in MQ polarization and reprogramming [30]. Association between chronic ECIG exposure and COPD was shown to be nicotine dependent [11] and altered lipid homeostasis in lungs can trigger pathogenesis of COPD [8]. Nicotine independent ECIG-induced lipid homeostasis was implicated in a mice model [7]. The study revealed aberrant phospholipids and increased surfactant-associated phospholipids in alveolar MQ and in airway. Chronic exposure of ECIG vapor on impaired response of MQ against viral infection as well as enhanced inflammation in lung, thus tissue damage was reported in that study [7]. In line with the findings of Madison et al. [7], our study suggests that ECIG-induced lipid laden macrophages (LLM) trigger inflammatory response in airways. However, LLM as a unique marker for EVALI has been questioned in a clinical case [6]. For instance, in the study, 7 out of 13 ECIG users were positive for LLM. Due to small sample size and interindividual differences, the study suggested further investigation with a bigger sample size whether LLM is a unique marker for EVALI or causes lung inflammation. M2 specific macrophage plays an important role in the inflammatory resolution process. We measured M1 and M2 specific pro and anti-inflammatory cytokines or chemokines both at gene and secreted protein level. IL-8 and IL-1 beta were upregulated both at secreted protein and gene level. Following ECIG exposure, M2 MQ specific surface marker CD206 expression was not affected significantly, but M2 specific cytokine IL-10 was decreased significantly, suggesting that ECIG exposure may not affect polarization towards M2 MQ, but the function of M2 MQ. We identified the existence of lipids, specifically phospholipids in ECIG liquid. Oxidized phospholipids induced pro-inflammatory effects have been reported in several studies [27, 31–33] but phospholipid-induced functional alteration in the airway is not well studied. A recent finding [34] showed that oxidized phospholipid induced oxidative stress and loss of viability in human epithelial cells BEAS-2B and Calu-3 cell lines, as well as epithelial cell barrier dysfunction, suggesting that phospholipids directly contribute to disease development. CD36 as a fatty acid transporter was reported to be involved in lipid induced M1 MQ polarization in diabetes [35, 36]. Wang et al. suggested that G9a potentially affects the expression of CD36 and lipid transportation, thus playing an important role for M1 MQ polarization [35]. In addition, lipid composition is associated with alveolar MQ polarization and low grade of inflammation in obese mice [35]. According to Prieur et al. [36] length and saturation of lipids affect MQ polarization. In obese mice M1 MQ specific marker was significantly higher in ECIG-exposed mice in comparison to control mice. Increased levels of cytotoxic lipids such as short chain fatty acid and more saturated lipids were suspected for M1 MQ polarization [36]. Our current study detected that ECIG induces CD36 expression and lipid accumulation as well as increased level of lipid peroxidation, indicating that CD36 mediated lipid accumulation from ECIG causes lipid peroxidation, which is a potential cause of M1 MQ polarization. The finding of Wang et al. and Prieur et al. is resembling the finding in our study. Furthermore, we validated the role of CD36 mediated lipid accumulation in M1 MQ polarization by silencing CD36, because CD36 silencing inhibited lipid accumulation and M1 MQ polarization.

Since MQ phenotypes decide the function of MQ, we investigated whether phagocytic activity of MQ was affected by exposure to ECIG. It has been shown that LLM has reduced phagocytic activity [33, 37]. Our experiment also revealed reduced phagocytosis of *E.coli* by MQ after ECIG exposure whereas CD36 silencing in MQ recovered the phagocytic activity of MQ significantly. Our findings are suggesting that lipid uptake or disrupted lipid homeostasis were causative factors of reduced phagocytosis of *E.coli* by altering MQ phenotype. TLR2 and TLR4 play an important role in innate immunity including pathogen recognition. In the MQ mediated immune response including phagocytosis of bacteria, TLR2 and TLR4 signaling is a hallmark in defense reaction and bacterial phagocytosis by MQ is promoted by TLR ligands [38, 39]. In our experiment TLR2 expression was not affected by ECIG-aerosol exposure but TLR4 expression was increased significantly after ECIG exposure. Ween et al. [10] detected reduced expression of TLR2 in response to ECIG in comparison to control condition. We observed that TLR2 expression is not affected by ECIG significantly. The study by Ween et al., used different flavors of ECIG with a higher nicotine concentration (18mg/ml) than the concentration we used in our experiments. Furthermore, the experiments were performed in THP1 derived macrophages under submerged condition. On the other hand, we used ECIG with (3mg/ml) or without nicotine, and primary MQ which was cultured at air–liquid interface. Therefore, due to different flavors of ECIG, higher concentration of nicotine or different cell types as well as exposure of ECIG-aerosol in culture medium in different experimental settings may explain non-identical outcomes in their study. However, the small sample size and limited technical replicates in our study is also a plausible reason for different results from the previous study. TLR2 and TLR4 promote and facilitate phagocytosis, but a wide range of cellular receptors and signaling such as CD35 and CD64 play a significant role in phagocytosis [35–37]. Often all these components jointly facilitate such activities but roles of TLR2, TLR4, CD35 and CD64 are distinct in phagocytosis. TLR2 and TLR4 facilitate phagocytosis indirectly by promoting recruitment of phagocytes. The role of the TLR2 and TLR4 in the actual engulfment of microorganisms by phagocytes is limited. CD35 directly contributes to the phagocytosis by facilitating recognition and uptake of compliment opsonized pathogen. CD35 acts as a receptor for complement components such as c3b which are deposited on the surface of microorganisms, thereby enhance phagocytosis activity. CD64 mediated phagocytosis is antibody dependent. Both CD35 and CD64 were downregulated in response to ECIG exposure, indicating that ECIG-induced lipid accumulation may affect those receptors and thus reduce uptake of *E.coli.* Since our cell culture condition was antibody free, therefore we speculate that CD35 reduction was a major cause of reduced phagocytosis. Moreover, reduced phagocytic activity by exposure to ECIG, can potentially increase the risk of infection. Incidence of pneumococcal pneumonia and pneumococcal nasopharyngeal colonization are increased in cigarette smokers [40, 41]. In addition, ECIG alters immune response against influenza infection and reduces survival from infection [7], furthermore, the role of lipid receptor in viral entry and replication has been implicated [42]. Our correlation analysis revealed that lipid accumulation was positively associated with lipid peroxidation, CD36 expression and M1 MQ polarization, on the other hand lipid accumulation was negatively correlated with the rate of phagocytosis and expression of the phagocytic receptors CD35 and CD64. Similarly, lipid peroxidation was also positively correlated with M1 MQ polarization whereas negatively correlated with the rate of phagocytosis and expression of the phagocytic receptors CD35 and CD64, which conforming our experimental findings that CD36 mediated lipid accumulation from ECIG triggers lipid peroxidation which is a potential causative agent for M1 MQ polarization and inflammation.

We conclude that ECIG exposure promotes polarization and function of MQ. Moreover, ECIG-induced altered lipid homeostasis affects phagocytic function of MQ. Our data contributes to explaining the possible link between ECIG exposure related lung injury and lipid-induced MQ phenotype and function.

### Supplementary Information

**Additional file 1.**

**Additional file 2.**

**Additional file 3.**

**Additional file 4.**

**Additional file 5.**

## Data Availability

Not applicable.
