# Supplementary figures and images for: Lipid from electronic cigarette-aerosol both with and without nicotine induced pro-inflammatory macrophage polarization and disrupted phagocytosis
Source: J Inflamm (Lond). 2023 Nov 17;20:39. doi: 10.1186/s12950-023-00367-6 (PMC10655339; doi:10.1186/s12950-023-00367-6)

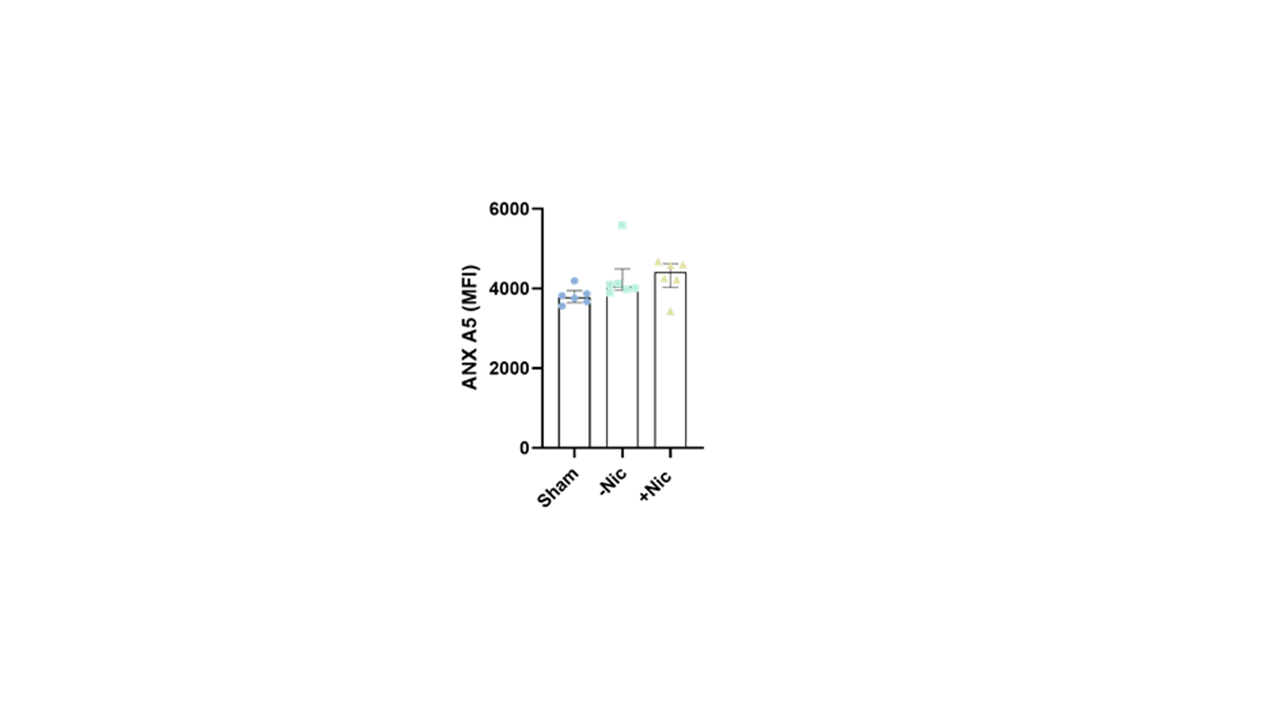

Supplement: Supplementary file 1 — Additional file 1. [file 12950_2023_367_MOESM1_ESM.tif]

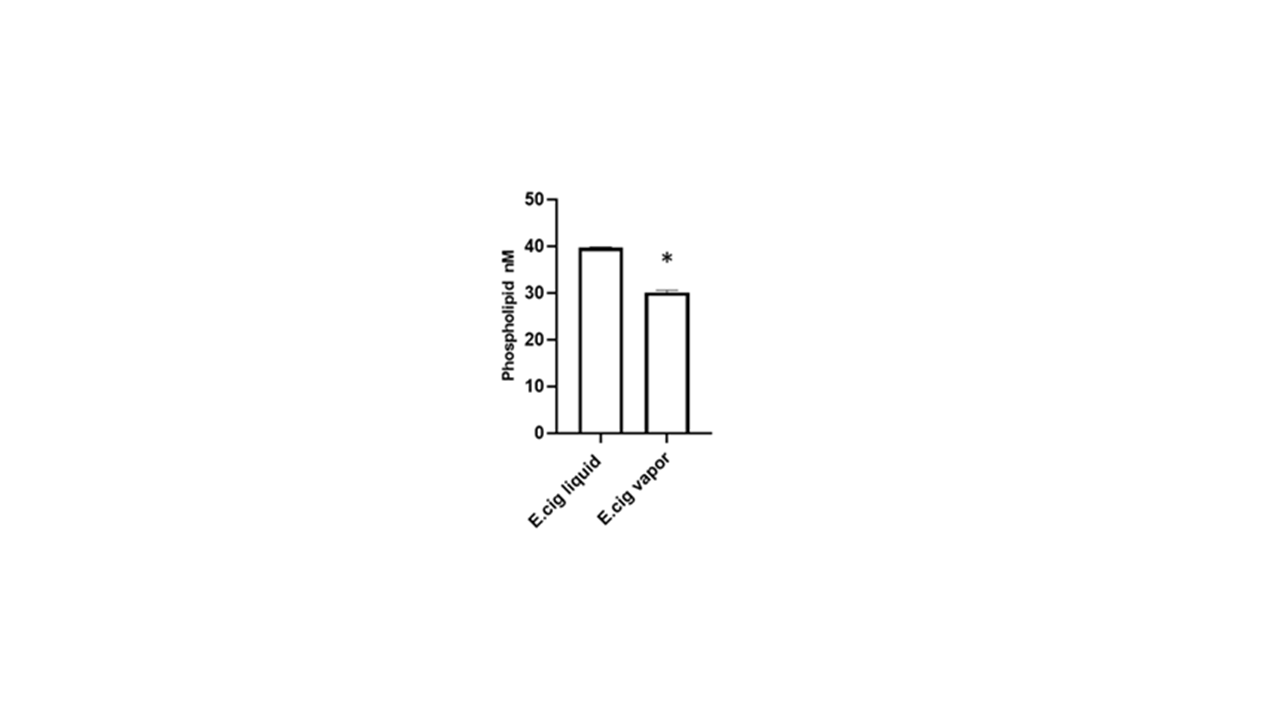

Supplement: Supplementary file 2 — Additional file 2. [file 12950_2023_367_MOESM2_ESM.tif]

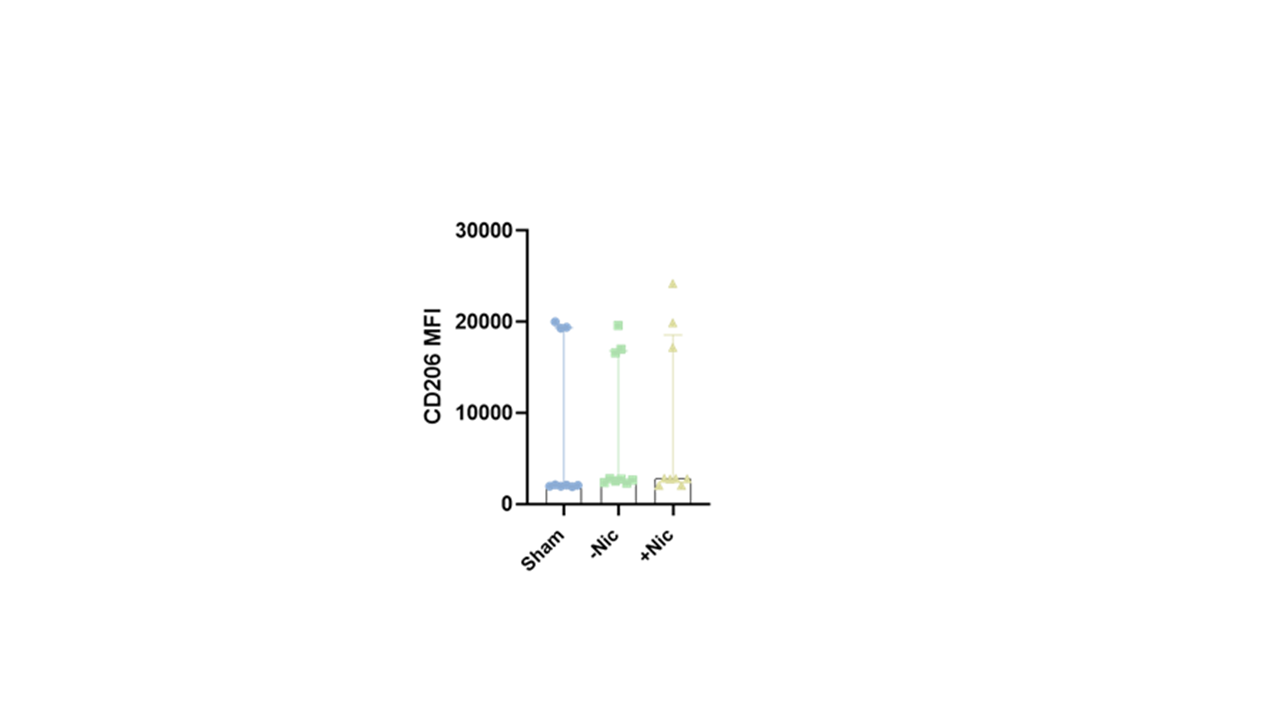

Supplement: Supplementary file 3 — Additional file 3. [file 12950_2023_367_MOESM3_ESM.tif]

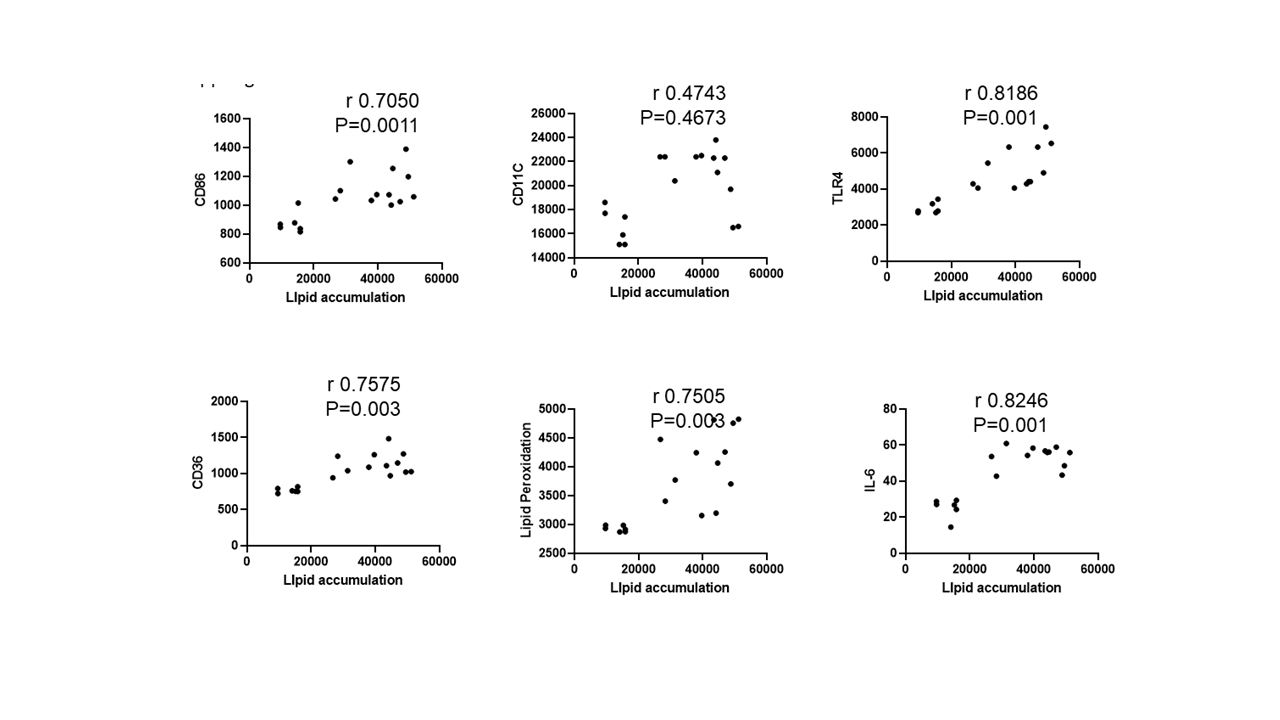

Supplement: Supplementary file 4 — Additional file 4. [file 12950_2023_367_MOESM4_ESM.zip › Supp figure 4 _(slide 1).TIF]

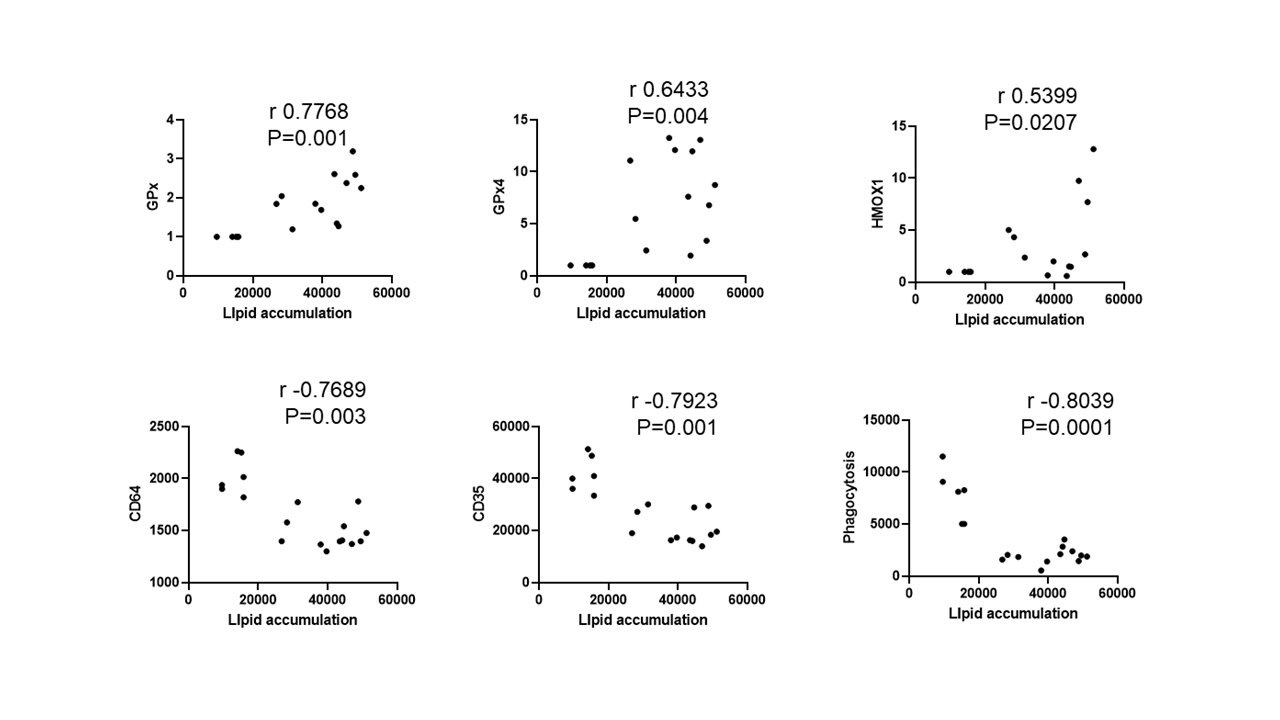

Supplement: Supplementary file 4 — Additional file 4. [file 12950_2023_367_MOESM4_ESM.zip › Supp figure 4 _(slide 2).TIF]

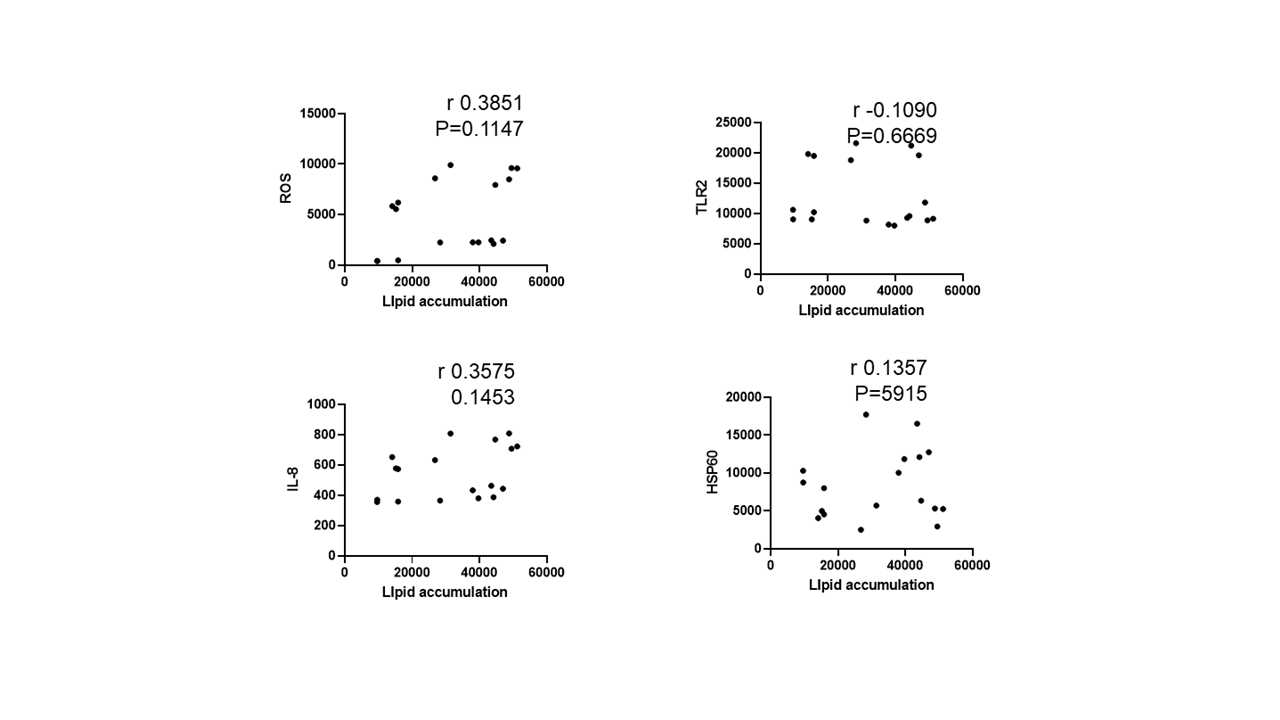

Supplement: Supplementary file 4 — Additional file 4. [file 12950_2023_367_MOESM4_ESM.zip › Supp figure 4 _(slide 3).TIF]

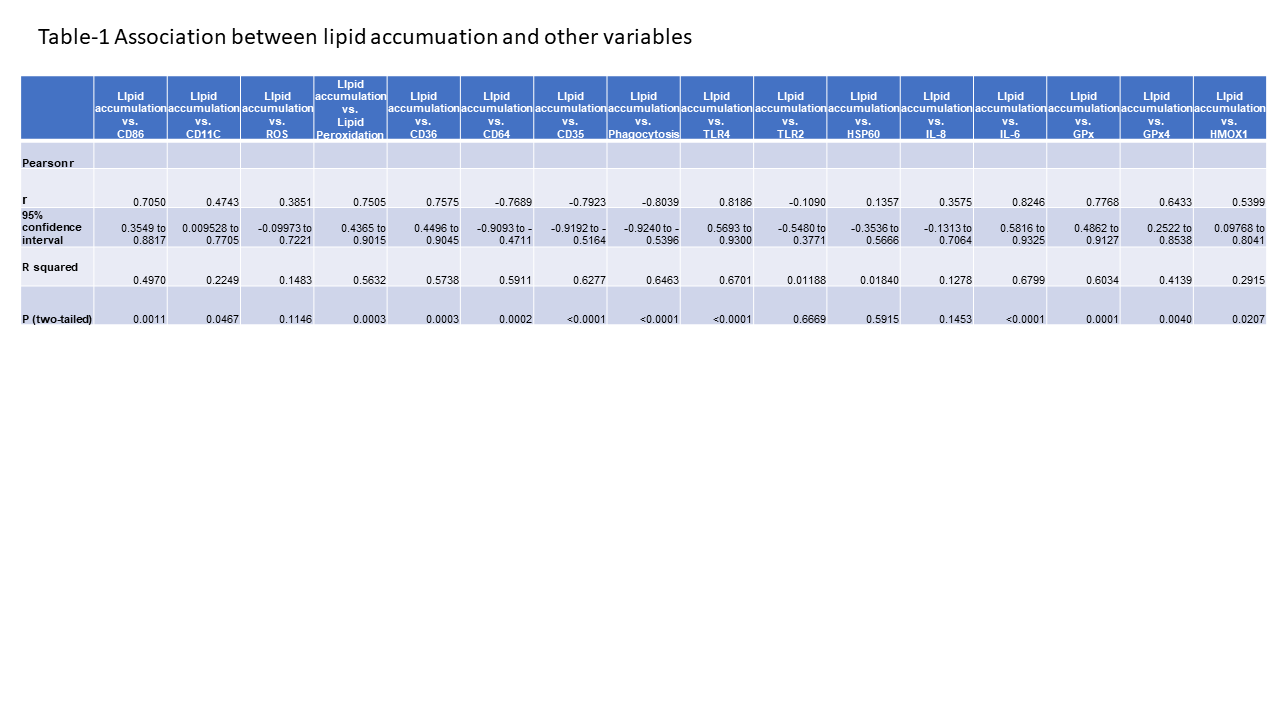

Supplement: Supplementary file 5 — Additional file 5. [file 12950_2023_367_MOESM5_ESM.tif]
